# Barbed Suture for Pancreaticojejunal Anastomosis: An Ally Against Pancreatic Fistula

**DOI:** 10.1002/jso.70048

**Published:** 2025-07-17

**Authors:** Rosita Sortino, Sophie M. Eschlboeck, Christoph Kuemmerli, Martin Bolli

**Affiliations:** ^1^ Clarunis University Center for Gastrointestinal and Liver Diseases, St. Clara Hospital and University Hospital Basel Basel Switzerland

**Keywords:** Pancreatectomy, pancreatic fistula, Pancreatic Neoplasms, pancreaticojejunostomy, Postoperative Complication

## Abstract

Pancreaticoduodenectomy (PD) is the standard treatment for resectable pancreatic head disease. The pancreatic anastomosis constitutes the mainstay of this procedure and is one of the drivers of its morbidity. Despite the development of a variety of techniques for pancreaticojejunostomy or pancreaticogastrostomy, high rates of postoperative pancreatic fistula (POPF), hampering early and fast patient recovery, are still reported. Here, based on the analysis of a cohort of 16 patients operated over 2 years, we describe a pancreaticojejunal anastomosis technique taking advantage of unidirectional barbed sutures. Although six of these patients were classified as “high risk,” according to updated International Study Group of Pancreatic Fistula (ISGPS) recommendations, we only observed one grade B POPF requiring antibiotic treatment, prolonged fasting and parenteral nutrition during the postoperative course. While larger studies are necessary to clarify this issue, our data suggests that barbed sutures could help prevent POPF development following PD.

## 1 Introduction

Pancreaticoduodenectomy (PD) is the standard treatment for malignant and benign pancreatic head disease [1]. Due to its technical complexity and potentially fatal complications, it remains one of the most challenging procedures in general surgery. While mortality has decreased to below 5% in recent decades, morbidity remains high, with rates approaching 50% [2, 3].

The Achilles' heel of PD is the development of postoperative pancreatic fistula (POPF), the most frequent and feared complication, with an incidence of 13% to 41% [4] and a mortality of up to 26% in severe cases [5]. The International Study Group of Pancreatic Fistula (ISGPF) first defined POPF in 2005 [6], and revised its criteria in 2016 to focus only on clinically relevant POPFs [7]. Despite various technical modifications proposed over the years, the optimal surgical approach to reduce POPF risk remains debated [8].

Among many factors influencing POPF formation, a key issue is the mechanical integrity of the pancreaticojejunal anastomosis [4, 5]. Conventional sutures can create uneven tension, particularly at knot sites, potentially increasing leak risk, especially in soft glands. Barbed sutures, which have shown benefits in other surgical fields [9], may offer a more even tension distribution, avoiding knot‐related stress. Their use in pancreatic surgery has been proposed in small series [10, 11, 12].

We report our experience with a unidirectional barbed suture (V‐Loc™) for pancreaticojejunal anastomosis, describe the technical details, and present a propensity score‐matched comparison with conventional sutures.

## 2 Materials and Methods

According to the most recent ISGPS classification, pancreatic texture and main pancreatic duct (MPD) diameter are among the key determinants of POPF risk [13].

**Figure 1:**
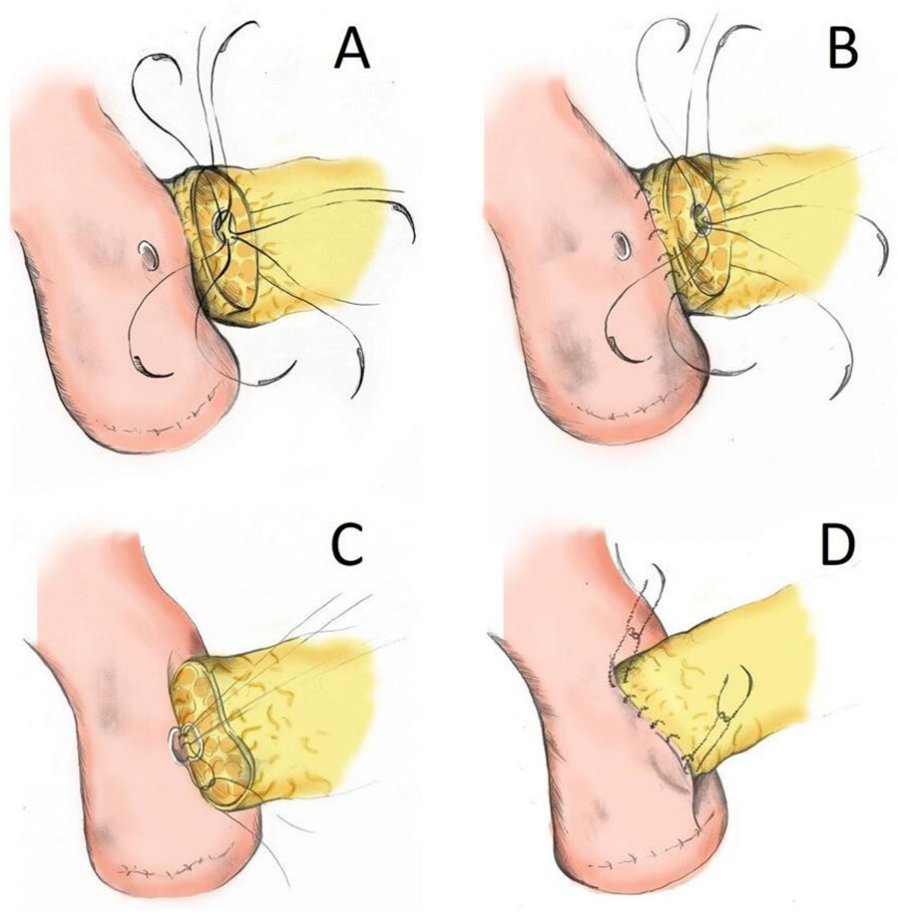
Schematic illustration of our surgical technique (A) Single‐armed 5.0 PDS sutures are placed starting at nine o'clock from the inside of the MPD and exiting on the transected surface of the pancreas. (B) V‐Loc^TM^ running suture is placed through the edge of the pancreatic stump and the seromuscular layer of the intestine to approximate the jejunum and pancreatic parenchyma. (C) The sutures previously placed in the MPD are passed through the entire thickness of the jejunal wall. (D) Finally, the anterior wall is sutured with another V‐Loc^TM,^ and both ends of the two barbed sutures are tied.

To explore whether the use of barbed sutures could help mitigate this risk, we retrospectively compared a modified duct‐to‐mucosa technique using a unidirectional barbed suture with a conventional interrupted suture approach, focusing on perioperative outcomes and clinically relevant POPF. Our barbed technique involved a modified duct‐to‐mucosa approach. A 5‐0 PDS stitch was placed at 9 o'clock within the MPD, exiting the pancreatic stump; four additional sutures were placed clockwise to 3 o'clock (Figure 1A). The posterior duct was sutured similarly. The external posterior layer was constructed with a running barbed suture (V‐Loc™; Covidien) along the pancreatic stump and jejunal seromuscular layer (Figure 1B). The loop was cut, and the ends retained for the anterior closure.

**Figure 2:**
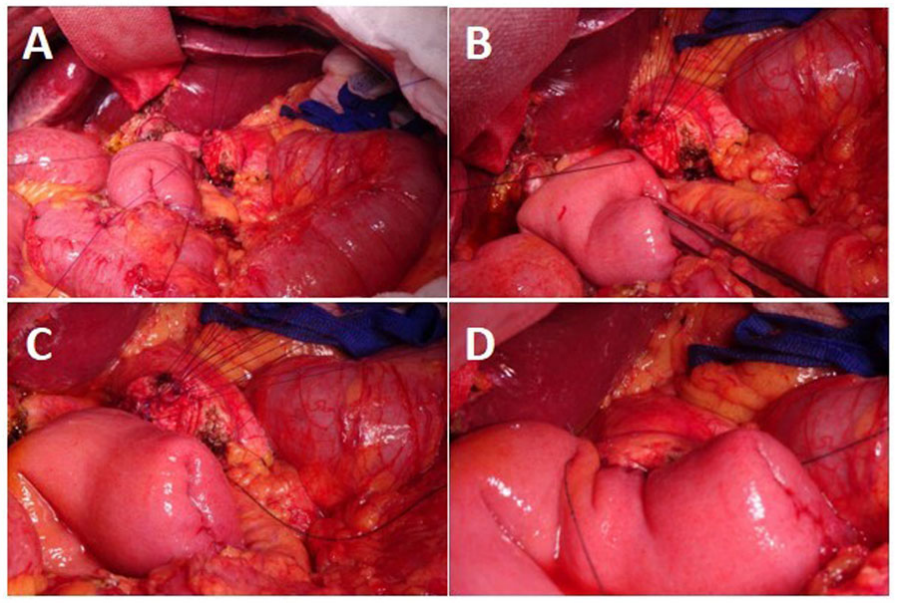
Intraoperative images illustrating key steps of pancreaticojejunostomy, as performed in our Institute (A) Suturing of the MPD. (B) Posterior placement of the first V‐Loc^TM^ running suture. (C) Approximation of pancreatic stump and jejunum. (D) Completion of the anterior wall of the anastomosis using the second V‐Loc^TM^ suture.

A jejunal opening was made opposite the MPD using electrocautery. Pre‐placed ductal sutures were passed through the full thickness of the jejunum, and a stent was inserted across the anastomosis. All sutures were then tied (Figure 1C). The anterior wall was completed using a second barbed suture (Figure 1D). Intraoperative steps are shown in Figure 2.

The conventional group underwent duct‐to‐mucosa anastomosis with interrupted non‐barbed sutures (PDS or Prolene), with seromuscular anchoring of the pancreatic capsule to the jejunum. All knots were tied manually.

Postoperatively, nasogastric tubes were removed on POD 7, and a contrast study was performed to assess for leakage. Somatostatin analogues were given until oral intake. Drains were managed per ISGPS guidelines, and amylase levels were measured on POD 3 and 5.

This study was approved by the Cantonal Ethics Committee (EKNZ; approval number 2023‐01201) and conducted in accordance with the Declaration of Helsinki. All patients provided informed consent.

## 3 Results

Sixteen patients (8 female, 8 male) underwent PD with barbed suture between January 2019 and January 2021. Median age was 75 years (IQR, 70.5–79.5), median BMI 25.5 kg/m² (IQR, 22.8–30.3). ASA classification: II (13%), III (81%), IV (6%). Table 1 summarizes patient data.

**Table 1:** Clinical‐pathological characteristics of treated patients (*n* = 16).

Sex	
Female	8 (50)
Male	8 (50)
Median Age, y	75 (IQR: 70.5–79.5)
ASA	
II	2 (13)
III	13 (81)
IV	1 (6)
Diagnosis	
PDAC	14
UC	2
Comorbidities	
Atrial Fibrillation	1
Obesity	3
Arterial Hypertension	8
Diabetes Mellitus	3
Dyslipidemia	3
Coronary heart disease	1
CKD	3
Pancreatic parenchyma	
Soft	6 (38)
Hard	10 (62)
MDP size, median	5 mm (IQR: 3–9)
FRS	
5	1
3	3
2	8
0	4

Abbreviations: ASA‐PS, American Society of Anesthesiologists‐Physical Status; CKD, chronic kidney disease; FRS, Fistula risk; MDP, main pancreatic duct; PDAC, pancreatic ductal adenocarcinoma, UC, undifferentiated carcinoma of the pancreas.

FRS identified four patients at moderate, eight at low, and four at negligible risk of POPF [14]. Six patients (38%) had soft pancreatic texture; six had MPD ≤ 3 mm. Based on ISGPS, six patients (38%) were high‐risk (Group C or D) [13]. Median operative time was 390 min (IQR, 360–420).

One patient (6%) developed a Grade B POPF, managed conservatively. One biochemical leak occurred. No reoperations or 30‐day mortalities. Other complications included one hemorrhagic shock, two cholangitis, one chyle leak, and one hepatic artery thrombosis.

A total of 38 patients were included in the final matched cohort: 14 in the barbed suture group and 24 in the conventional suture group. Baseline characteristics were adequately balanced between groups, with standardized mean differences < 0.2.

The use of barbed sutures was associated with a significantly lower overall complication rate compared to conventional sutures (46.2% vs. 83.3%, *p* = 0.047). The median Comprehensive Complication Index (CCI) was also significantly reduced in the barbed suture group compared to the conventional group (median 0.00 vs 29.6, *p* = 0.030), indicating a meaningful reduction in cumulative postoperative morbidity.

Clinically relevant POPF occurred in one patient (7.1%) in the barbed suture group and in four patients (16.7%) in the conventional group (*p* = 0.402). A nonsignificant trend toward shorter ICU stay (56.0 vs. 72.0 h, *p* = 0.402) and length of hospital stay (19.5 vs. 28.0 days, *p* = 0.102) was also observed in the barbed suture group.

## 4 Discussion

The performance of the pancreaticojejunal anastomosis is a critical factor in determining outcomes following pancreaticoduodenectomy (PD). Technical failure or impaired healing of this anastomosis can lead to the development of postoperative pancreatic fistula (POPF), one of the most feared and frequent complications in pancreatic surgery [5, 13, 14, 15],[16]. A soft pancreatic texture and a narrow main pancreatic duct (MPD) are well‐established risk factors for fistula formation, particularly when excessive tension or manipulation compromises the integrity of the pancreatic remnant [14].

In such cases, meticulous surgical technique becomes paramount to mitigate these inherent risks. Precise duct‐to‐mucosa suturing with uniform tension, minimal tissue trauma, and careful handling of the fragile pancreatic parenchyma are essential to promote optimal healing and reduce leakage. The adoption of innovative suture materials and techniques, such as barbed sutures, may help overcome these challenges by providing consistent tension distribution and eliminating knot‐related stress points, thus potentially improving anastomotic integrity even in high‐risk pancreatic tissue.

Barbed sutures, such as the V‐Loc™ system, represent a promising technical solution due to their knotless nature and capacity for uniform tension distribution across the anastomosis. The elimination of repeated knot‐tying may reduce traction injury and preserve microvascular perfusion in the pancreatic parenchyma. In our initial cohort of 16 patients undergoing PD with barbed suture for pancreaticojejunal anastomosis, only one patient (6%) developed a clinically relevant POPF, despite several being classified as moderate‐to‐high risk based on current ISGPS criteria. No 30‐day mortalities were observed, and the complication profile was favorable overall.

The utility of barbed sutures has been demonstrated in various surgical disciplines—including gynecologic, urologic, and gastrointestinal surgery—where they have been associated with reductions in operative time, improved hemostasis, and fewer complications [9, 10, 11, 12],[17,18]. In pancreatic surgery, however, their use remains limited, and evidence supporting their clinical benefit is still emerging.

## 5 Conclusions

The V‐Loc™ barbed suture appears to be a safe and effective option for the construction of pancreaticojejunal anastomosis during pancreaticoduodenectomy. In our initial cohort, the incidence of clinically relevant postoperative pancreatic fistula (POPF) was low, even among patients with risk factors for anastomotic leakage.

While the reduction in clinically relevant POPF did not reach statistical significance, the overall complication profile and trend toward shorter hospitalization suggest a potential clinical benefit.

**Figure 3:**
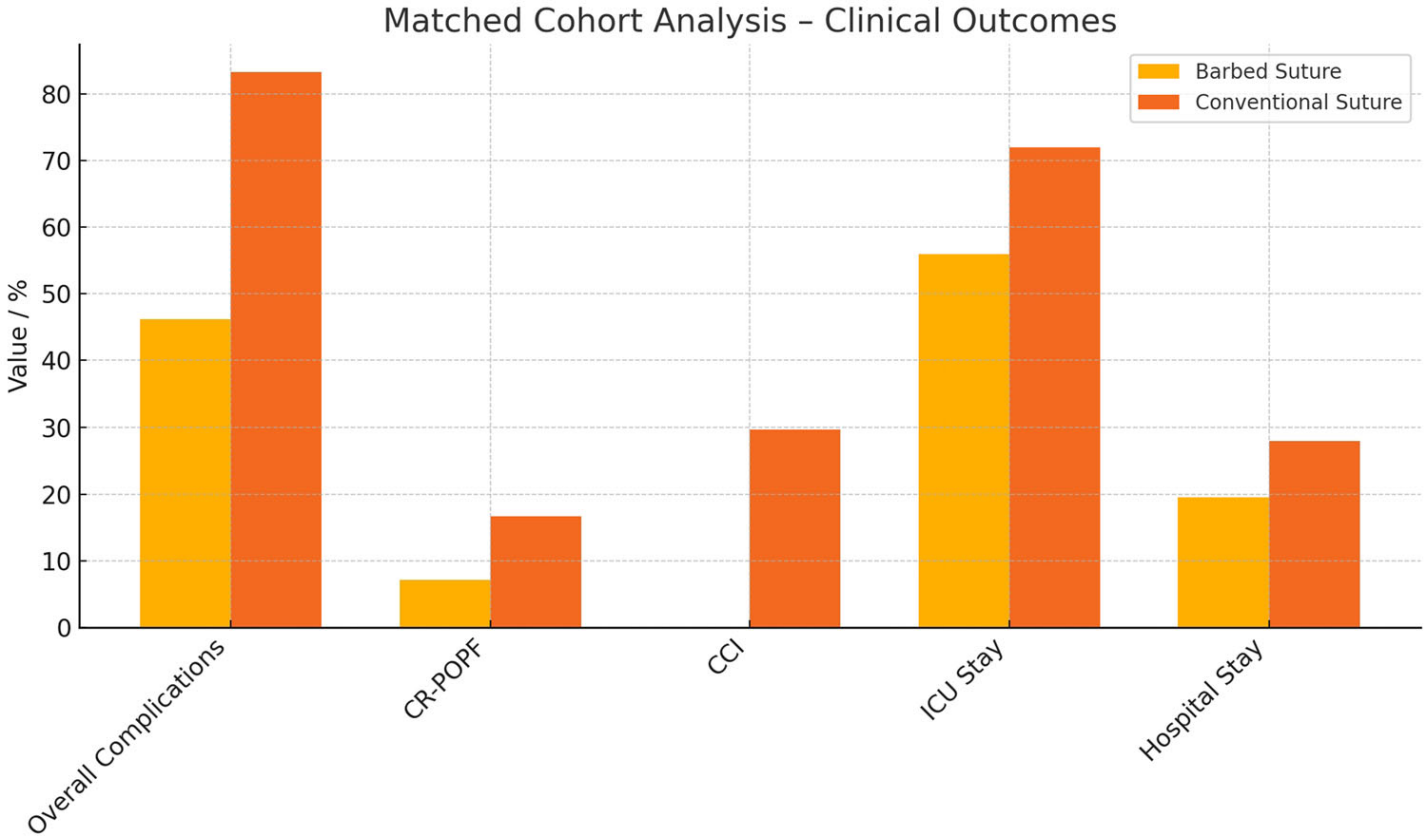
Matched cohort analysis of clinical outcomes following pancreaticojejunal anastomosis using barbed versus conventional sutures.

These findings support the role of barbed sutures as a valuable technical adjunct in pancreatic surgery. Further prospective studies are warranted to confirm these preliminary results and explore their impact on long‐term outcomes. (Figure 3)

## Ethics Statement

This study was approved by the Cantonal Ethics Committee: Ethikkommission Nordwest‐ and Zentralschweiz – EKNZ (approval number 2023‐01201).

## Conflicts of Interest

The authors declare no conflicts of interest.

## Synopsis

Clinical‐pathological characteristics.

## Data Availability

The data that support the findings of this study are available from the corresponding author upon reasonable request.
